# DEVELOPMENT OF PODCAST IN SERVICE OF THE NEXT GENERATION OF GEROPSYCHOLOGISTS

**DOI:** 10.1093/geroni/igac059.1976

**Published:** 2022-12-20

**Authors:** Branden Schaff, Rachel Weiskittle, Lindsey Jacobs Dodson

**Affiliations:** University of Colorado Colorado Springs, Colorado Springs, Colorado, United States; University of Colorado Colorado Springs, Colorado Springs, Colorado, United States; University of Alabama Tuscaloosa, Tuscaloosa, Alabama, United States

## Abstract

The current geropsychology workforce falls woefully short of meeting the mental healthcare needs of older adults, with only 3% of licensed psychologists identifying Geropsychology as their primary or secondary specialty. Formal training in geriatric mental health is extremely limited. Out of the over 400 APA-accredited doctorate-level psychology programs in the U.S., only five provide specialty training in Geropsychology. Consequently, the vast majority of psychologists who treat older adults have never received formal education or training in geriatrics. It is imperative that evidence-based geropsychology education is disseminated to a wide audience of mental health providers across all levels of training and career development. The Geropsychology Podcast was developed to address this gap as a novel pedagogical resource commensurate with educational competencies established by the ‘Pikes Peak Model for Professional Geropsychology.’ Topics include geriatric assessment methods, intervention, consultation, and foundational knowledge on adult development and aging. The feasibility and acceptability of the podcast was evaluated with a two-year pilot study from 2019 to 2021. During this time, eleven episodes were published across multiple podcast platforms. Preliminary results found the episodes were downloaded by over 1,500 unique listeners across 34 countries. These results indicate that The Geropsychology Podcast is scalable, acceptable, and potentially fulfilling an educational need that people are actively seeking. This poster describes the podcast’s model of curriculum development and ongoing quality metrics and impact analysis. Future work holds that this podcast will continue to be a vital resource for the field.

